# Exploring the Impact of Biological Agents on Protecting Against Experimental Periodontitis: A Systematic Review of Animal-Based Studies

**DOI:** 10.1155/bmri/1716735

**Published:** 2024-12-02

**Authors:** Gabriela Ezequiel Oliveira, Davi da Silva Barbirato, Bruna Silva de Menezes, Milenna Silva Fuly, Henrique Cassebe Ledo Pelegrine, Debora Caliendo Bonilha, Julia Gabrielle Pereira de Alencar, Leticia Helena Theodoro, Rafael Scaf de Molon

**Affiliations:** ^1^Department of Diagnosis and Surgery, School of Dentistry, São Paulo State University-UNESP, Aracatuba, São Paulo 16015-050, Brazil; ^2^Department of Basic and Oral Biology, Faculty of Dentistry of Ribeirão Preto, University of São Paulo (FORP/USP) 14040-904, Ribeirão Preto, São Paulo, Brazil; ^3^Division of Periodontics, Dental School, Federal University of Rio de Janeiro-UFRJ, Rio de Janeiro, Rio de Janeiro 21941-617, Brazil

**Keywords:** alveolar bone, animal model, bone resorption, periodontal disease, periodontitis

## Abstract

**Aim:** This systematic review was aimed at addressing the focused question: What is the protective potential of biological agents against alveolar bone resorption during the progression of experimental periodontitis (EP)?

**Material and Methods:** The study protocol was registered in the Open Science Framework database (doi:10.17605/OSF.IO/3P2HY). A comprehensive literature search was conducted across PubMed, Web of Science, Cochrane Library, Scopus, and Embase databases up to December 2023. Inclusion criteria consisted of preclinical studies in animal models of EP that examined the effects of biological agents on preventing periodontal bone loss and reducing tissue inflammation. Studies were excluded if they (i) used non-EP animal models; (ii) focused on antimicrobial agents; (iii) centered on prebiotics or probiotics; (iv) evaluated compounds not classified as biologicals; or (v) included randomized clinical trials, clinical studies, or reviews. Eligibility was determined based on the PI/ECOs framework, and study quality was assessed using the SYRCLE risk-of-bias tool.

**Results:** After screening an initial pool of 5236 records from databases, registries, and hand searches, 39 studies met the inclusion criteria. A total of 23 biological agents were evaluated across these studies. The majority of studies employed the ligature-induced model of EP to test the effectiveness of biologicals as preventive or therapeutic interventions. The dosage of biological agents and the duration of disease induction varied depending on the EP model. In all studies, the main outcome—alveolar bone loss, a hallmark of EP—was significantly inhibited by biological agents, which also reduced proinflammatory mediators when compared to untreated controls. A key strength of this review is the high number of studies included, most of which were classified as having low risk of bias. However, a notable limitation is the absence of a meta-analysis, the short follow-up periods in the included studies, and the heterogeneity among the compound dosages and route of administration.

**Conclusion:** This systematic review demonstrates that biological agents are effective in reducing bone loss and mitigating inflammation during EP progression. Randomized clinical trials are needed to confirm these findings in human populations.

## 1. Introduction

Periodontitis (PD), characterized by chronic inflammation of the supporting tissues around the teeth, develops due to a complex interaction between the host and parasites, progressively compromising the integrity of the periodontal tissues [1, 2]. It is characterized by bacterial-induced inflammatory responses and destruction of periodontal tissues, including the periodontal ligament, cement, and alveolar bone. The more severe stages of PD (Stages III and IV) affect over 700 million people, representing approximately 11% of the global population [3]. Indeed, PD ranks as the sixth most prevalent chronic condition globally and is considered the leading cause of tooth loss in adults [4, 5]. Consequently, PD presents a significant public health challenge due to its high prevalence and the substantial burden caused by tooth loss and impaired chewing function, negatively impacting the quality of life.

An increasing body of research has linked PD with systemic conditions such as diabetes mellitus [6–10], cardiovascular diseases [11, 12], and rheumatoid arthritis [13, 14], through the dissemination of pathogenic bacteria and their products into the bloodstream [14–17]. Briefly, the biological connection between PD and systemic diseases is supported by the understanding that low-grade chronic inflammation, triggered by the host immune response to bacterial biofilm at the gingival margin, can potentially affect distant organs [18–21]. PD may exacerbate systemic diseases through several mechanisms: (i) bacterial invasion: The immune system responds to bacterial infiltration by releasing proinflammatory cytokines, for example, tumor necrosis factor-*α* (TNF-*α*), interleukin (IL)-1*β*, and IL-6, and other mediators to eliminate the microbial threat. However, prolonged inflammation can lead to immune dysregulation, causing systemic effects that extend beyond the oral cavity; (ii) systemic inflammatory burden: The continuous release of inflammatory mediators into the bloodstream during PD increases the overall inflammatory burden on the body. This heightened inflammation can contribute to the development of various diseases, particularly those driven by inflammatory processes, such as cardiovascular diseases, diabetes mellitus, and rheumatoid arthritis; and (iii) bacterial translocation and bacteremia: Pathogenic bacteria associated with PD—such as *Porphyromonas gingivalis*, *Treponema denticola*, and *Tannerella forsythia*—can enter the bloodstream, especially during activities like toothbrushing or chewing in individuals with periodontal conditions. Once these pathogens and their endotoxins (e.g., lipopolysaccharides (LPSs)) enter the systemic circulation, they may affect distant organs and contribute to the development of systemic diseases [18–21]. As a result, the treatment of PD plays a crucial role in reducing both local and systemic inflammations.

PD is characterized by a unique property in which tissue damage arises from the host's innate and adaptive immune response to the multiple microorganisms associated with periodontal biofilm [1, 4]. Imbalance between the resident commensal microbiota and the host response leads to the dysbiosis of the oral microbiome, and thus, increased bacterial load and persistent inflammatory process are the hallmark of disease [22]. In individuals susceptible to PD, the host immune response is typically exacerbated, dysregulated, and destructive, resulting in the infiltration of immune cells and inflammatory cells (macrophages and neutrophils) [23]. Innumerous proinflammatory cytokines such as IL-1, IL-6, IL-17, prostaglandin E2 (PGE2), TNF-*α*, and matrix-degrading enzymes (cathepsin and matrix metalloproteinases (MMPs)) produced by lymphocytes, fibroblast, leukocytes, and epithelial cells have been identified as key molecules inducing tissue destruction, and the expression of these molecules is significantly increased during disease progression [23]. More specifically, these cytokines facilitate the increased activation of the receptor activator of nuclear factor kappa B ligand (RANKL) pathway in osteoblasts, fibroblasts, or lymphocytes. This ultimately leads to the differentiation and activation of osteoclasts, resulting in the destruction of mineralized connective tissue [24]. Therefore, the regulation of the immune response and the inhibition of osteoclastogenesis are key factors that should be considered when testing potential new treatment modalities for PD.

According to the clinical practice guideline from the European Federation of Periodontology, the recommendations for treating Stage I–III PD are based on four distinct interventions [25]. The initial step of therapy involves behavioral changes and the control of supragingival biofilm, gingival inflammation, and associated risk factors. The second step of therapy is aimed at controlling both supra- and subgingival biofilms and calculus through subgingival instrumentation, with the goal of managing the infection and halting the inflammatory process. Subgingival instrumentation may involve adjunctive local or systemic medications. The third step involves treating areas that are unresponsive to the second step and may include various types of periodontal surgical interventions. Finally, supportive periodontal care should be implemented to prolong the benefits and maintain periodontal stability over time [25].

The nonsurgical periodontal therapy (NSPT), the term used to describe subgingival instrumentation, is accomplished through scaling and root planning (SRP), the gold standard treatment for Stage I–III PD [25]. The use of adjunctive therapies to treat PD relies on the premise that not all the individuals respond well to NSPT especially in noncompliant and susceptible patients and also in patients with systemic comorbidities (diabetes) associated with the presence of common risk factors for disease progression, such as local factors (deep periodontal pockets and complex root anatomy), environmental factors (smoking), and genetic background (polymorphisms) [23]. Therefore, the use of adjunctive therapies (biological) aiming at modulating the destructive events of the innate and adaptive immune host response has been proposed in preclinical and clinical studies as a potential therapeutic strategy to treat PD targeting inflammatory mediators and bone-resorbing osteoclasts [2, 23].

In the past years, a growing number of studies investigating the beneficial effects of adjunctive therapies in clinical trials aiming at decreasing inflammation and alveolar bone destruction and improving the outcomes of NSPT alone have been performed [26–29]. Moreover, different animal models of PD have been used to test the efficacy of different compounds and the molecular mechanisms involved in the inhibition of bone resorption [24, 30, 31]. Therefore, the current systematic review sought to investigate the evidence on the beneficial effects of biological agents against inflammation and bone resorption in experimental periodontitis (EP).

## 2. Materials and Methods

### 2.1. Objectives

This systematic review was aimed at addressing the focused question: What is the protective potential of biological agents against alveolar bone resorption during the progression of EP?

### 2.2. Protocol and Registration

This manuscript was performed at the Systematic Review Facility (SyRF) [32], as recommended by the Collaborative Approach to Meta-Analysis and Review of Animal Experimental Studies (CAMARADES). The construction of this manuscript follows the guidelines outlined in the PRISMA 2020 statement [33]. The review project was created using the SyRF platform, and the protocol was registered in the Open Science Framework (OSF) database under the DOI number 10.17605/OSF.IO/3P2HY.

### 2.3. Focused Question

The focused question is constructed on the PICOD norm.

– Population (P): animal models with EP
– Intervention (I): biological factors utilized or examined for the prevention or control of alveolar bone resorption in EP. Biological factors encompass endogenously synthesized compounds that impact biological processes not otherwise categorized under enzymes, hormones, or hormone antagonists
– Comparator (C): sham or placebo group
– Outcome of interest (O): main outcomes: (i) prevention/control of alveolar bone resorption by biological factors and (ii) effect of biological factors on alveolar bone, including cellular, molecular, tissue, and functional assessment––additional outcomes: (i) harmful consequences, adverse events, and systemic impact of biological factors used/tested for prevention/control of bone resorption in experimental P; (ii) benefit-to-harm ratio of biological factors used/tested for prevention/control of bone destruction in EP; (iii) mechanistic insights of biological factors used/tested for prevention/control of alveolar bone resorption in EP; and (iv) hierarchy of biological factors with greater effectiveness (prevention/control of alveolar bone resorption), safety (harmful consequences, adverse events, and systemic impact), evidence (study quality and RoB assessment), and lack of evidence from preclinical studies to support Phase I studies (clinical trial design) on the subject
– Primary study design (D): preclinical study (in vivo) referring to tests, experiments, and procedures that researchers perform in or on a laboratory animal; this study was aimed at answering the main question: What is the protective potential of biological factors against alveolar bone resorption in EP?

### 2.4. Study Selection Criteria and Exclusion Criteria

Only preclinical studies conducted in vivo on the protective potential of biological factors against alveolar bone resorption in EP were included. Studies were excluded if they (i) utilized an animal model of noninduced PD; (ii) involved antimicrobial agents; (iii) focused on prebiotics and probiotics; (iv) investigated compounds not classified as biological; (v) lacked available evidence correlating to the intervention or outcomes; (vi) had inaccessible full-text versions; (vii) review papers, opinion articles, and conference abstracts; or (viii) randomized or nonrandomized clinical trials and cohort, cross-sectional, and longitudinal clinical studies. No restrictions were imposed on the language or publication date.

### 2.5. Information Sources

An all-encompassing literature search was accompanied across five databases: MEDLINE via PubMed, Web of Science (WOS) retrieved through Clarivate Analytics, Cochrane Central Register of Controlled Trials (CENTRAL), Embase, and Scopus through Elsevier. Grey literature sources were accessed throughout Google Scholar and the System for Information on Grey Literature in Europe (SIGLE) via OpenGrey. Hand searching was performed in specialized periodicals. Additionally, specialists were recognized by expertscape.com and communicated for additional record bases.

### 2.6. Search Strategy

For search strategies, databases were incorporated into MeSH terms, entry terms, and keywords to inquire WOS, PubMed, Cochrane Library, and grey literature, as well as protocol records. For Embase and Scopus databases, additional search terms such as Entree, Index, and DeCS/MeSH terms were utilized. All terms were combined using Boolean operators “OR” and “AND” to connect key concepts in a “building blocks” strategy (refer to Table 1). Electronic searches were conducted in December 2022, and database alerts were set up to identify studies published after the search date, up until the manuscript submission process.

### 2.7. Selection Process

The recovered manuscripts were transferred to the Rayyan reference manager, where duplicates were excluded both automatically by the program (based on perfect match) and manually. The assortment procedure was accompanied in two stages: Stage 1 involved three investigators (B.S.M., M.S.F., and R.S.M.) who individually assessed the titles and abstracts of all recovered references, employing the inclusion principles. In Stage 2, the same three investigators individually applied the exclusion criteria during the full-text screening. The complete manuscripts were assessed and considered in their entirety. Inter-reviewer reliability in the assortment procedure was determined using the Cohen *κ* test, with a threshold value set at 0.80 [34], as described. Any disagreements at any stage were solved through discussion and shared decision-making with a third reviewer (D.S.B.). The conclusive assessment was based on a thorough reading of the entire text.

### 2.8. Data Collection Process

Data extraction was individually performed by the three reviewers (B.S.M., M.S.F., and R.S.M.) using a standardized form. In instances of disagreement, consensus was reached through discussion with a fourth reviewer (DSB). The qualitative results were discussed and described in the article during a consensus meeting, following the order of the PICO elements: study (preclinical studies where EP was induced in the animals according to the experimental method used, that is, ligature-induced bone loss, LPS injection, or oral inoculation of bacteria), population (animals such as rat and mouse), intervention (biologicals used to manage EP, which could be administered via locally or systemically), comparator (placebo), combination therapies, alveolar bone resorption (as the main study outcome), and secondary findings (immunoinflammatory profile). Adverse effects were also evaluated as secondary outcomes. Authors were contacted via email over five uninterrupted weeks, as necessary, to achieve further information on study methods and clarification of data such as missing or unclear information regarding dosages, route of administration, frequency of application, experimental time frame, and number and strain of animals used.

### 2.9. Study Risk-of-Bias (RoB) Assessment

Three reviewers (B.S.M., M.S.F., and R.S.M.) independently assessed the quality of the included preclinical studies using the SYRCLE's RoB tool for animal studies [35]. This instrument, derived from the Cochrane RoB tool, has been tailored to address biases specific to animal intervention studies. The SYRCLE's tool comprises 10 fixed domains of bias and also six types of biases, as previously described [35]. A decision of “yes” specifies a low RoB, “no” denotes a high RoB, and “unclear” denotes unsatisfactory aspects for appropriate bias evaluation. The RoB analysis was conducted independently and blinded. In instances of disagreement, consensus was reached through discussion with a fourth reviewer (D.S.B.).

### 2.10. Synthesis Methods

The process of selecting studies, the characteristics of the studies, the evaluation of bias in the studies, the outcomes of singular manuscripts, the outcomes of combining results, the identification of reporting biases, and the level of certainty in the evidence are explained using textual descriptions, visuals, and tables. The aggregation of qualitative findings followed the guidelines outlined in the SWiM reporting protocol [36].

### 2.11. Reporting Bias Assessment

Confounding factors such as animal model; experimental protocol; biological factors; and method to assess alveolar bone resorption and cellular, molecular, tissue, and functional outcomes were considered in the result synthesis. The occurrence of publication bias was investigated as described previously [37, 38].

## 3. Results

### 3.1. Study Selection

**Figure 1:**
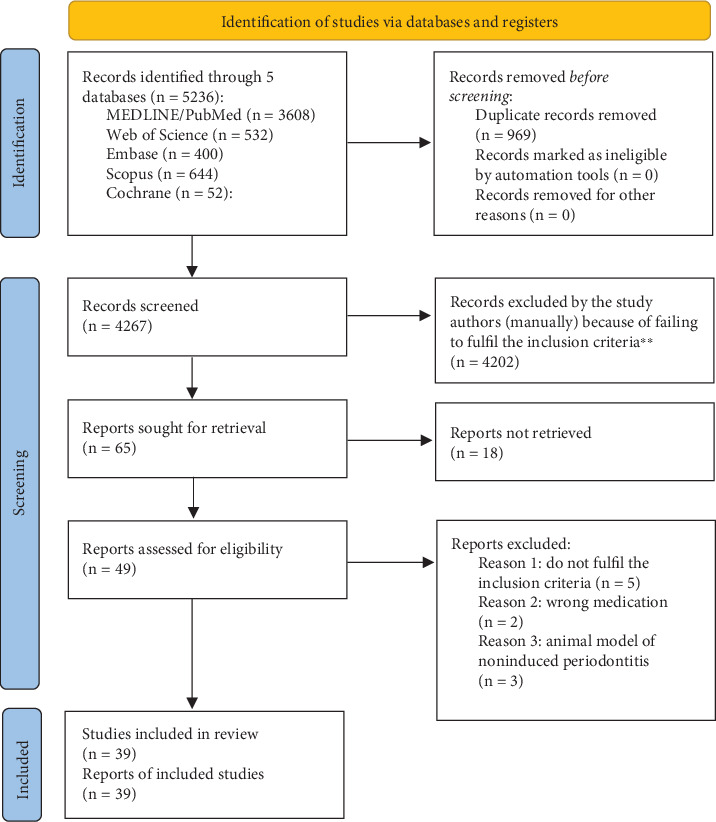
Screening and enrolment according to the PRISMA flow diagram. ⁣^∗^Consider, if feasible to do so, reporting the number of records identified from each database or register searched (rather than the total number across all databases/registers). ⁣^∗∗^If automation tools were used, indicate how many records were excluded by a human and how many were excluded by automation tools. *Source:* Page et al. [33].

Figure 1 demonstrates a flowchart that summarizes the identification of study selection via databases and registers of studies that assessed the beneficial effects of biological agents on experimental PD. Following database screening, 5236 studies were identified by the authors, which were carried out into five databanks (PubMed, Scopus, Embase, Cochrane, and WOS). After the removal of duplicates (969), the search strategies identified 4267 possible eligible articles, of which 4202 were immediately excluded after reading the titles and the abstracts. The full text of the 65 remaining manuscripts was assessed, and 18 articles were then excluded, as they did not fulfill the eligibility criteria, totaling 49 studies. During the process for study eligibility, 10 studies were excluded after reading the full text due to wrong animal model and wrong medication used. Therefore, 39 manuscripts were included to serve as the basis for this systematic review.

### 3.2. Study Characteristics

The 39 included reports were published between 2000 and 2023 (Table 2). Different animal models were used in those studies, comprising mainly rodents. Ten studies have used Wistar rats as an animal model [39–42, 48, 49, 61, 66, 71, 77]; 14 studies [43, 50, 56–60, 62, 63, 67, 69, 72, 74, 75] have used C57BL/6 mice; eight studies [44–46, 51, 54, 55, 68, 70] employed Sprague–Dawley rats; one study [47] have used *Macaca fascicularis* as an animal model; two studies [52, 53] used the F344 inbred rats; one study [65] used BALB/c as a mouse model; two studies [73, 76] have used the DIO mouse model (C57BL/6 background); and finally, one study [64] did not report the mouse strain utilized.

Most of the included studies utilized a ligature-induced EP model, totalizing 23 articles [39–45, 48–53, 55, 56, 61, 63, 65, 66, 70, 73, 74, 77] varying in different thread materials, such as silk, cotton, and dental floss. LPS injection as an EP model was used in three studies [46, 54, 68], in which Cirelli et al. [46] used LPS from *P. gingivalis*, while Kirkwood et al. and Rogers et al. used LPS from *Aggregatibacter actinomycetemcomitans* [54, 68]; ligatures soaked with *P. gingivalis* as an animal model were also used in four studies [47, 62, 75, 76]. Oral bacterial inoculation with live *P. gingivalis* was used in five studies [57–59, 69, 72], oral inoculation with *A. actinomycetemcomitans* was used in three studies [60, 64, 67], and one study [71] has used *P. gingivalis* plus *F. nucleatum* to induce EP via oral inoculation.

Studies using the ligature-induced bone loss have applied the therapeutic approach in six reports [40–42, 55, 70, 77] (treatment starting after the disease onset), while another 33 studies have used preventive strategies against alveolar bone destruction using the ligature model (treatment started immediately after ligature placement) or oral inoculation of periodontopathogenic bacteria or LPS injection. Additionally, eight studies have utilized local treatment (injection on the gingival tissue) [42, 43, 47, 50, 65, 70, 74, 77], and the other included reports used the systemic administration of biologicals to treat EP by means of intraperitoneal injection [39, 40, 52, 55–59, 62, 63, 66, 67, 72], oral gavage [41, 54, 64, 68, 73], subcutaneous injection [44, 45, 48, 51, 53, 60, 61, 70, 75], intramuscular injection [46], intravenous injection [49], adoptive cell transfer [69], biological dissolved in the drinking water [71], and finally systemic infusion [77]. The follow-up period of evaluation in the included studies ranged from 5 days to 8 weeks.

The key biological agents evaluated in these studies were as follows: the humanized monoclonal anti-human IL-6 receptor (tocilizumab) [39]; melatonin, a hormone synthetized in the pineal gland and other organs, was tested in four studies [40, 55, 66, 71]; vitamins K_2_ [41] and D_3_ [41, 58, 59, 72] tested in another four studies; the second-generation concentrate named injectable platelet-rich fibrin (i-PRF) [42] and an autologous hemoderivate material obtained by the disruption of platelets; the platelet lysate [77]; an immunoregulatory cytokine with anti-inflammatory properties, IL-35 [43]; a protein secreted primarily by osteocytes that regulates osteoblast-mediated bone formation named sclerostin (sclerostin monoclonal antibody (Scl-Ab)) [44, 70]; a major regulator of bone remodeling and calcium homeostasis, a protein composed of 84 amino acids, the parathyroid hormone (PTH) [45, 53, 61]; TNF-*α* blockers (TNFR:Fc—monoclonal anti-TNF-*α* antibodies or fusion proteins containing p75 TNFR linked to the Fc portion of human IgG1) [46]; the soluble IL-1 receptor Type 1 (sIL-1R1) that functions as a competitive inhibitor of IL-1 [47]; the nerve growth factor (NGF)–neutralizing antibody [48]; infliximab, a chimeric, human IgG1 TNF-*α* monoclonal antibody [49, 52]; CD40, a membrane-associated protein and a member of the TNF receptor superfamily [50]; the human recombinant OPG fusion protein (rhOPG-Fc) [51, 75]; a competitive inhibitor of p38*α* mitogen-activated protein kinase (MAPK), SD282 [54, 68]; a human monoclonal antibody that bind to RANKL, a TNF-super family cytokine produced by osteoblasts and stromal cells in bone tissues (anti-mouse RANKL monoclonal antibody) [56, 75]; sRAGE, which binds ligand and blocks interaction with cell-surface RAGE [57]; the BET (bromodomain and extraterminal domain) inhibitor JQ1 (a cell-permeable small molecule) [62]; the cytotoxic T lymphocyte–associated antigen 4 (CTLA-4) [63]; the soluble epoxide hydrolase inhibitor (she) TPPU [64]; the IL-17A-neutralizing antibody [65]; met-RANTES (specific antagonist of CCR1 and CCR5 receptors) [67]; B10 cells [69]; a novel adiponectin (APN) receptor agonist (AdipoAI) [73] and APN [76]; and finally, the Toll-like receptor 9 (TLR9) agonist cytidine–phosphatase–guanosine oligodeoxynucleotide (CpG), CD40 ligand (CD40L) [74].

CD40L is a member of the TNF receptor superfamily expressed on CD4 T cells. It interacts with CD40 on antigen-presenting cells, leading to various biological effects. The interaction between CD40 and CD40L is critical for B cell differentiation and activation, promoting the production of IL-10 [78, 79]. TLRs, which act as pattern recognition receptors, are expressed by B cells, enabling the detection of pathogen-associated molecular patterns and playing an essential role in both innate and adaptive immune responses [80]. Previous studies have shown that B cells in the spleen serve as a key reservoir of IL-10, particularly when stimulated by TLR9 and its CpG agonist [81].

### 3.3. Outcomes of Interest

The majority of the included studies have utilized morphometric analysis [49, 50, 57, 58, 60, 62, 64, 67, 69, 72, 74, 76] and microcomputed tomography (micro-CT) [39, 42–46, 51, 54, 56, 63, 65, 68, 70, 73, 77] to quantitate the bone-level changes, an important parameter to assess periodontal status. Morphometric analyses were accomplished determining the distance between the cement–enamel junction (CEJ) to the alveolar bone crest (ABC) (2D linear measurements) after staining the jawbones with methylene blue, using specific software as reference to perform the measurements. A more accurate method of analysis, the micro-CT, is able to quantitate the tridimensional volumetric alterations and the architectural parameters of bone. One study employed scanning electronic microscopy (SEM) [59], two studies used conventional 2D radiography [40, 48], and seven studies utilized histological analysis to measure alveolar bone loss [41, 47, 52, 53, 55, 66, 75]. Histomorphological measurements using histologic images allow quantitation of bone destruction through linear measurements or by analyzing the total area of bone loss in the furcation or in the interproximal area. Two studies [61, 71] did not quantify the amount of bone loss inhibition after experimental PD treatment. It is noteworthy to mention that almost all studies have demonstrated that biological agents sheltered the host from the aggravation of alveolar bone destruction when compared to the control groups, except by one study [41] that used vitamin K_2_, D_3_, or a combination of both. Taken together, based on the presented results, the biological agents are considered a safe adjunctive treatment of EP (without side effects) to be used as a systemic or local compound. Furthermore, all of them showed encouraging data regarding prevention and treatment of EP.

Furthermore, gene expression analysis (RT-qPCR) was accomplished by 15 studies to examine the effects of biological agents on the expression levels of proinflammatory cytokine markers, such as IL-1*β*, TNF-*α*, IL-6, IL-17, IL-10, INF-*γ*, RANKL, and OPG [39, 43, 46, 48, 50, 52, 60–62, 65, 67, 69, 73, 74, 77]. Immunohistochemistry (IHC) analysis and TRAP staining were performed by 21 studies [42, 45, 46, 48–57, 59, 61, 63, 68, 69, 72, 74, 76] to investigate the effects of biologicals on protein levels, especially RANKL, OPG, and MMP-9, and on the number of positive-stained osteoclasts. Biochemical analysis of serum was performed by three studies [40, 41, 55] mainly to investigate the levels of TRAP-5b (marker for osteoclast) and bone formation markers, such as osteocalcin (OCN) and C-terminal telopeptide of Type 1 collagen (CTx-1). ELISA was made by another 12 studies [41, 43, 44, 49, 57, 58, 60, 66, 67, 69, 70, 72] to investigate protein levels in serum, and finally, western blot analysis was accomplished by seven studies [49, 58, 59, 64, 66, 72, 73] to investigate the phosphorylation of signaling pathways involved in bone resorption, such as Janus family kinase 1 (JAK1), nuclear factor kappa-light-chain-enhancer of activated B cells (NF-*κ*B)-p65, and signal transducer and activator of transcription (STAT)-3. Collectively, the main outcomes of the selected studies regarding diminishing of bone loss and decreased expression of proinflammatory cytokines as well as inhibition of signaling pathways phosphorylation were achieved in all studies suggesting a promising alternative to use as an adjuvant approach to treat EP.

### 3.4. Quality Assessment of Evidence

The assessment of evidence of the included studies was considered following the ARRIVE guidelines checklist containing a 21 items, and the scores varied from 14 to 19 (Tables 3(a), 3(b), and 3(c)). None of the included articles reported information regarding animal inclusion or exclusion criteria. Only two studies reported data access (where study data are available) [53, 63]. Additionally, just four studies [39, 52, 63, 68] described the sample size calculation for study power, and information about conflict of interest was described in 11 studies [42–45, 53, 63, 65, 69, 71, 73, 77]. Almost half of the included studies reported the randomization process of experimental groups [39–43, 45, 55, 58, 59, 63, 66, 71, 72, 74–77], and 17 studies did not provide information about the blinding process during the analysis [42, 44, 45, 49, 50, 53, 55–57, 62, 66, 67, 69, 70, 73, 74, 76, 77]. No information was found in 24 articles on animal care [47, 48, 50, 51, 53, 54, 56, 57, 59–62, 64–70, 73–77]. Overall, all the articles were supported by strong study design, outcome measures, statistical methods, interpretation of scientific evidence, generalizability translation, protocol registration, experimental animals and procedure, results, abstract, background, objectives, and ethical statement.

### 3.5. RoB Within Studies

**Figure 2:**
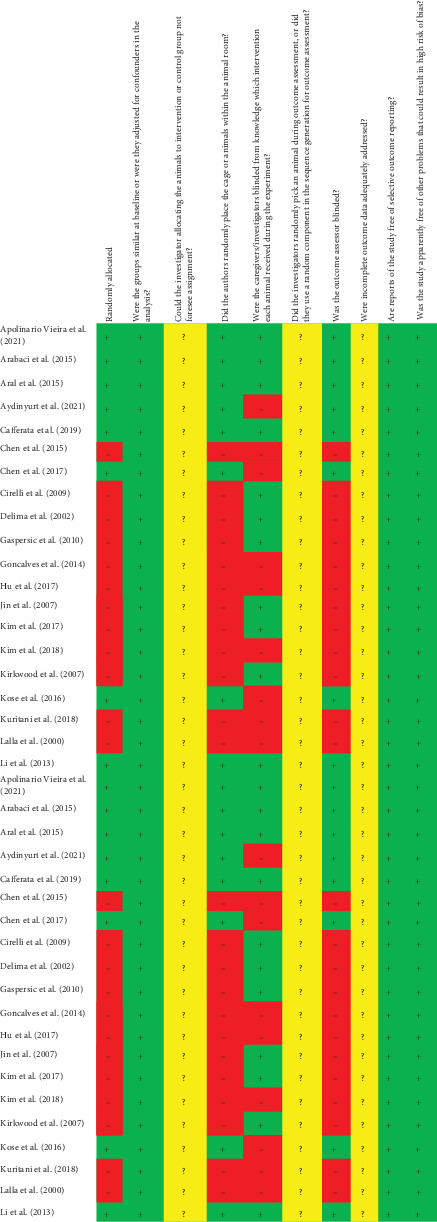
Risk of bias in the included manuscripts according to the SYRCLE checklist tailored for preclinical studies.

To evaluate the RoB in the included manuscripts, the SYRCLE checklist tailored for preclinical studies was employed. As depicted in Figure 2, the majority of included studies performed well across most domains and checklist items, although some deficiencies were noted. It is noteworthy that domains such as “allocation concealment,” “random outcome assessment,” and “attrition bias–incomplete outcome data” were all rated as unclear in the included studies. Additionally, in 22 studies, the sequence generation (random allocation of animals to treatments) and random outcome assessment were not adequately described [44, 46–54, 56, 57, 60–62, 64, 65, 67–70, 73]. Taken together, 17 articles were categorized as having low RoB [39–43, 45, 55, 58, 59, 63, 66, 71, 72, 74–77], 15 reports were classified to have moderate RoB [44, 46–48, 51–54, 60, 61, 64, 65, 67, 68, 73], and seven studies were considered to have high RoB [49, 50, 56, 57, 62, 69, 70].

## 4. Discussion

According to the Food and Drug Administration, a biological agent encompasses various therapeutic substances such as viruses, therapeutic serum, toxins, vaccines, blood components or derivatives, allergenic products, proteins, or similar products, all intended for the prevention, treatment, or cure of human diseases or conditions [82]. In the context of PD, the term “biologic” is more narrowly defined as a therapeutic agent with biological activity aimed at inhibiting alveolar bone loss, the hallmark of PD [83]. Thus, this comprehensive review was aimed at assessing the beneficial effects of some biological agents on EP. Founded on the main outcomes of the selected studies, biological agents have demonstrated efficacy as host modulators of the inflammatory response and strong inhibitors of alveolar bone destruction in various animal models, whether employed preventively or therapeutically. Their potential to halt alveolar bone resorption positions them as promising therapeutic compounds for the treatment of periodontal disease. Consequently, a recent consensus statement on the use of biologicals in clinical practice [84] affirmed that biologics are generally safe for use in periodontal practice and offer additional benefits when combined with conventional periodontal treatment.

Animal models are extensively employed to replicate experimental conditions in various human-related diseases. By utilizing experimental models of PD, researchers gain insight into the molecular mechanisms underlying the immunopathogenesis of chronic inflammatory diseases [85, 86]. The ligature-induced bone loss, oral inoculation, and LPS injection EP models, which were the primary models utilized in the included studies, contribute significantly to our understanding of the events that lead to either protection or tissue damage as a consequence of bacterial dysbiosis and the dysregulation of the host immune response [85]. Animal models of EP simulate an infection and inflammatory process parallel to that observed in humans, thereby mimicking the natural disease pathogenesis. Rodent models of EP offer several advantages, including cost-effectiveness for scientific research, ease of handling, and relatively low costs. Additionally, the physiological systems and anatomical structures of rodents are well understood, further enhancing the utility of these models [87]. Importantly, these animal models can also play a pivotal role in the development of more successful therapeutic strategies. They aid in hypothesis validation and demonstrate the effectiveness of new treatment therapies, thereby supporting decisions regarding human clinical research [88, 89].

Systematic reviews of preclinical studies are widely acknowledged for their significance in identifying interventions with the greatest preventive or therapeutic potential, paving the way for testing in randomized clinical trials. These reviews offer robust and comprehensive descriptions of animal studies, aiding in informed decision-making [90]. Ensuring the reliability and quality of included manuscripts is essential in this process. In the present systematic review, the ARRIVE checklist, which consists of 21 items, was utilized to assess the quality and reliability of the included manuscripts [91]. Achieving scores between 14 and 19 in the quality assessment indicates that these reports were deemed reliable and of high quality.

The included studies in this systematic review demonstrated innumerous methodological variations regarding the EP induction models (ligature, oral gavage, LPS inoculation), animals used (mouse and rat), inoculation of bacteria (*P. gingivalis*, *F. nucleatum*, and *A. actinomycetemcomitans*), materials for ligature (cotton, silk, and dental floss), number of days of for disease induction, and treatment duration with the biologicals (Table 2). Following a comprehensive analysis of the characteristics of each included manuscript, it was observed that the most commonly used animal strain was the C57BL/6 mouse, and the predominant EP model employed was ligature-induced bone loss, typically placed around the first and second maxillary molars in the majority of cases. The primary methods utilized for bone loss evaluation were micro-CT and histological analyses. Despite the inconsistency of the models encountered in the literature, limited reports have investigated and matched the sequential progression, as well as the local and systemic outcomes of EP, across diverse biological regimens.

The hallmark of PD is the alveolar bone resorption that happens in consequence of the imbalance between the microbial insult and the host immune response. Therefore, inhibition of bone loss was set as the main outcome parameter in this systematic review. Quantitation of alveolar bone resorption in the included studies was performed by means of morphometric analysis [49, 50, 57, 58, 60, 62, 64, 67, 69, 72, 74, 76], micro-CT [39, 42–46, 51, 54, 56, 63, 65, 68, 70, 73, 77], conventional radiograph [40, 48], SEM [59], and histomorphometric analysis [41, 47, 52, 53, 55, 66, 75]. Except by one study [41] that fails to show inhibition of bone loss after treatment and by two studies that did not quantify the resorption of the alveolar bone [61, 71], all the other studies showed remarkable ameliorative effects regarding inhibition of bone loss. Osteoclasts, the cells accountable for resorbing the bone tissue, were evaluated in several studies [42, 45, 46, 48–57, 59, 61, 63, 68, 69, 72, 74, 76] utilizing the TRAP staining analysis. Decreased osteoclast activity and osteoclast number after biological treatment seem to significantly regulate alveolar bone resorption in those studies. Most of the biological investigated prevented alveolar bone loss by suppressing RANK-L-mediated osteoclast development decreasing the ratio of RANKL/OPG.

In this current systematic review, the systemic administration of biologicals appeared to effectively inhibit alveolar bone destruction in the different animal models used [39–41, 44–46, 48, 49, 51–64, 66–73, 75, 77], as well as the local treatment [42, 43, 47, 50, 65, 70, 74, 77]. Both local and systemic administrations of biologicals have shown promise in controlling systemic and local inflammation provoked by bacteria. However, local treatment, involving direct injection into the gingival tissue, appears to be a more practical and reliable approach for treating PD in humans compared to systemic administration. This is because the compound is applied directly into the periodontal pocket or sulcus, thereby enhancing its local effects against inflammation-induced bone loss. Of importance, some studies included showed that biologicals are effective in ameliorate systemic conditions associated with PD, such as diabetes mellitus [45, 52, 53, 55, 57, 58, 72, 73], obesity [71, 76], and ovariectomized osteoporosis animal models [44] evidencing that these compounds have direct actions on the systemic inflammatory response of others inflammatory (diabetes and obesity) and noninflammatory (osteoporosis) conditions. Therefore, further investigations are warranted to explore the influence of these biologicals in other systemic inflammatory conditions in clinical trials.

This systematic review included 39 studies that met the inclusion criteria, and these relatively high numbers of studies strengthen the achieved findings and support the use of biologicals in preclinical studies. When analyzing the data for RoB according to the SYRCLES's checklist specific for preclinical studies, most of the included reports demonstrated a low risk [39–43, 45, 55, 58, 59, 63, 66, 71, 72, 74–77] or moderate risk [44, 46–48, 51–54, 60, 61, 64, 65, 67, 68, 73] of bias. The studies classified as having moderate and high RoB were correlated with missing information regarding attrition bias, allocation concealment, and sequence generation. Consequently, we can infer that the studies included in this review are, most of them, of quality, reproducible, and reliable studies.

It is important to acknowledge several limitations of our study. First, this review included only animal studies, making it difficult to directly extrapolate the potential benefits of these biological agents to humans. Additionally, the studies reviewed exhibited significant variability in terms of medication dosage, route of administration, and treatment duration. This variability complicates efforts to determine precise dosages and treatment timelines for future clinical trials. Besides, the included studies employed different PD models, each with distinct disease progression and severity, further contributing to variability in the findings. Some of the studies failed to report adverse effects, which can limit the guiding for future research and clinical practice. Finally, while the RoB was found to be low or moderate, several studies lacked adequate details regarding allocation concealment, sequence generation, and blinding processes. These shortcomings may influence the reliability of the results. This heterogeneity could potentially affect the generalizability and reliability of the findings, making it difficult to draw definitive conclusions about the overall effectiveness of specific biological agents.

It is also important to underscore the need for randomized clinical trials to validate the promising results observed in animal models. Since all the studies included were carried out in animals, it is difficult to translate them to humans. Moreover, the differences in disease mechanisms between animals and humans necessitate cautious interpretation of the results. The transition from preclinical to clinical studies is crucial for confirming the safety and efficacy of these biological agents in human subjects.

## 5. Conclusion

Collectively, the outcomes of this study demonstrate that the use of biological agents presents a promising alternative for treating EP, whether applied systemically or locally. These biological molecules possess the unique ability to halt the progression of bone loss and decrease the inflammatory process without causing additional side effects, making them attractive potential agents for preventing and treating EP. While the results are promising, further investigation through randomized clinical trials is warranted to evaluate the success of biologicals in humans.

## Figures and Tables

**Table 1:** Search strategies.

**Search strategy**	**Electronic databases**
(“biological products” OR “biological factors” OR “products, biological” OR “biological product” OR “product, biological” OR “biologic product” OR “product, biologic” OR “biologic products” OR biopharmaceuticals OR biopharmaceutical OR biological OR biologic OR “biological drug” OR “drug, biological” OR “biologic drugs” OR “drugs, biologic” OR “biological medicine” OR “medicine, biological” OR “biological medicines” OR “medicines, biological” OR biologicals OR “biologic medicines” OR “medicines, biologic” OR “biologic pharmaceuticals” OR “pharmaceuticals, biologic” OR biologics OR “biologic drug” OR “drug, biologic” OR “biological drugs” OR “drugs, biological” OR “natural products” OR “natural product” OR “product, natural” OR “organic chemicals” OR “chemicals, organic” OR “organic chemical” OR “chemical, organic” OR “biological products therapeutic use” OR “biologic factors and agents acting on the immune system” OR “natural products and their synthetic derivatives”) AND (“periodontal diseases” OR periodontitis OR “chronic periodontitis” OR “aggressive periodontitis” OR “periodontitis, aggressive, 2” OR periodont⁣^∗^ OR parodont⁣^∗^ OR “pyorrhea alveolaris” OR pericement⁣^∗^ OR “experimental periodontitis”) AND (“bone resorption” OR “bone resorptions” OR “resorption, bone” OR “resorptions, bone” OR “osteoclastic bone loss” OR “bone loss, osteoclastic” OR “bone losses, osteoclastic” OR “loss, osteoclastic bone” OR “losses, osteoclastic bone” OR “osteoclastic bone losses” OR osteoclasts OR “alveolar bone loss” OR “alveolar bone losses” OR “alveolar process atrophy” OR “alveolar process atrophies” OR “alveolar resorption” OR “alveolar resorptions” OR “resorption, alveolar” OR “resorptions, alveolar” OR “bone loss, periodontal” OR “bone losses, periodontal” OR “periodontal bone losses” OR “periodontal bone loss” OR “periodontal resorption” OR “periodontal resorptions” OR “resorption, periodontal” OR “alveolar bone atrophy” OR “alveolar bone atrophies” OR “bone atrophies, alveolar” OR “bone atrophy, alveolar” OR “bone loss, alveolar” OR “experimental bone loss”) NOT (“dental implants” OR “implant, dental” OR “implants, dental” OR “dental implant” OR “dental implants, mini” OR “dental implant, mini” OR “mini dental implant” OR “mini dental implants” OR “dental prostheses, surgical” OR “dental prosthesis, surgical” OR “surgical dental prostheses” OR “surgical dental prosthesis” OR “prostheses, surgical dental” OR “prosthesis, surgical dental” OR “dental implantation” OR “tooth, artificial”) NOT (review OR reviews OR systematic review[Publication type] OR “systematic reviews as topic” OR “systematic reviews” OR “systematic review” OR meta-analysis[Publication type] OR “meta-analysis as topic” OR “meta-analysis” OR “meta-analyses” OR “meta analysis” OR “meta analyses” OR overview OR “umbrella review”)	MEDLINE|PubMed

(“biological products” OR “biological factors” OR biopharmaceuticals OR biological OR biologic OR “biological drug” OR “biological medicine” OR “biologic medicines” OR “biologic pharmaceuticals” OR “biologic drug” OR “biological drugs” OR “natural products” OR “natural product” OR “organic chemicals” OR “organic chemical” OR “biological products therapeutic use” OR “biologic factors and agents acting on the immune system” OR “natural products and their synthetic derivatives”) AND (“periodontal diseases” OR periodontitis OR “chronic periodontitis” OR “aggressive periodontitis” OR “periodontitis, aggressive, 2” OR periodont⁣^∗^ OR parodont⁣^∗^ OR “pyorrhea alveolaris” OR pericement⁣^∗^ OR “experimental periodontitis”) AND (“bone resorption” OR “bone resorption” OR “osteoclastic bone loss” OR osteoclasts OR “alveolar bone loss” OR “alveolar bone losses” OR “alveolar process atrophy” OR “alveolar process atrophies” OR “alveolar resorption” OR “alveolar resorption” OR “periodontal bone losses” OR “periodontal bone loss” OR “periodontal resorption” OR “periodontal resorption” OR “alveolar bone atrophy” OR “alveolar bone atrophies” OR “experimental bone loss”)	Web of Science, Cochrane CENTRAL, LILACS|VHL, and other sources

(ALL (“biological AND products” OR “biological AND factors” OR “products, AND biological” OR “biological AND product” OR “product, AND biological” OR “biologic AND product” OR “product, AND biologic” OR “biologic AND products” OR biopharmaceutical OR biopharmaceutical OR biological OR biologic OR “biological AND drug” OR “drug, AND biological” OR “biologic AND drugs” OR “drugs, AND biologic” OR “biological AND medicine” OR “medicine, AND biological” OR “biological AND medicines” OR “medicines, AND biological” OR biologicals OR “biologic AND medicines” OR “medicines, AND biologic” OR “biologic AND pharmaceuticals” OR “pharmaceuticals, AND biologic” OR biologics OR “biologic AND drug” OR “drug, AND biologic” OR ”biological AND drugs” OR “drugs, AND biological” OR “natural AND products” OR “natural AND product” OR ”product, AND natural” OR “organic AND chemicals” OR “chemicals, AND organic” OR “organic AND chemical” OR “chemical, AND organic” OR “biological AND products AND therapeutic AND use” OR “biologic AND factors AND agents AND acting AND on AND the AND immune AND system” OR “natural AND products AND their AND synthetic AND derivatives”)) AND (ALL (“periodontal AND diseases” OR periodontitis OR “chronic AND periodontitis” OR “aggressive AND periodontitis” OR “periodontitis, AND aggressive, AND 2” OR periodont⁣^∗^ OR parodont⁣^∗^ OR “pyorrhea AND alveolaris” OR pericement⁣^∗^ OR “experimental AND periodontitis”)) AND (ALL (“bone AND resorption” OR “bone AND resorption” OR “resorption, AND bone” OR “resorption, AND bone” OR “osteoclastic AND bone AND loss” OR “bone AND loss, AND osteoclastic” OR “bone AND losses, AND osteoclastic” OR “loss, AND osteoclastic AND bone” OR “losses, AND osteoclastic AND bone” OR “osteoclastic AND bone AND losses” OR osteoclasts OR “alveolar AND bone AND loss” OR “alveolar AND bone AND losses” OR “alveolar AND process AND atrophy” OR “alveolar AND process AND atrophies” OR “alveolar AND resorption” OR “alveolar AND resorption” OR “resorption, AND alveolar” OR “resorption, AND alveolar” OR “bone AND loss, AND periodontal” OR “bone AND losses, AND periodontal” OR “periodontal AND bone AND losses” OR “periodontal AND bone AND loss” OR “periodontal AND resorption” OR “periodontal AND resorption” OR “resorption, AND periodontal” OR “alveolar AND bone AND atrophy” OR “alveolar AND bone AND atrophies” OR “bone AND atrophies, AND alveolar” OR “bone AND atrophy, AND alveolar” OR “bone AND loss, AND alveolar” OR “experimental AND bone AND loss”))	Scopus

#1 “biological products” OR “biological factors” OR “products, biological” OR “biological product” OR “product, biological” OR “biologic product” OR “product, biologic” OR “biologic products” OR biopharmaceuticals OR biopharmaceutical OR biological OR biologic OR “biological drug” OR “drug, biological” OR “biologic drugs” OR “drugs, biologic” OR “biological medicine” OR “medicine, biological” OR “biological medicines” OR “medicines, biological” OR biological OR “biologic medicines” OR “medicines, biologic” OR “biologic pharmaceuticals” OR “pharmaceuticals, biologic” OR biologics OR “biologic drug” OR “drug, biologic” OR “biological drugs” OR “drugs, biological” OR “natural products” OR “natural product” OR “product, natural” OR “organic chemicals” OR “chemicals, organic” OR “organic chemical” OR “chemical, organic” OR “biological products therapeutic use” OR “biologic factors and agents acting on the immune system” OR “natural products and their synthetic derivatives” #2 ”periodontal diseases” OR periodontitis OR “chronic periodontitis” OR “aggressive periodontitis” OR “periodontitis, aggressive, 2” OR periodont⁣^∗^ OR parodont⁣^∗^ OR “pyorrhea alveolaris” OR pericement⁣^∗^ OR “experimental periodontitis” #3 “bone resorption” OR “bone resorption” OR “resorption, bone” OR “resorption, bone” OR “osteoclastic bone loss” OR “bone loss, osteoclastic” OR “bone losses, osteoclastic” OR “loss, osteoclastic bone” OR “losses, osteoclastic bone” OR “osteoclastic bone losses” OR osteoclasts OR “alveolar bone loss” OR “alveolar bone losses” OR “alveolar process atrophy” OR “alveolar process atrophies” OR “alveolar resorption” OR “alveolar resorption” OR “resorption, alveolar” OR “resorption, alveolar” OR “bone loss, periodontal” OR “bone losses, periodontal” OR “periodontal bone losses” OR “periodontal bone loss” OR “periodontal resorption” OR “periodontal resorption” OR “resorption, periodontal” OR “alveolar bone atrophy” OR “alveolar bone atrophies” OR “bone atrophies, alveolar” OR “bone atrophy, alveolar” OR “bone loss, alveolar” OR “experimental bone loss” #1 AND #2 AND #3	Embase

**Table 2:** Summary of the study characteristics.

**Author/year**	**Animal model**	**Intervention/comparison groups**	**Type of biological therapy**	**Follow-up time**	**Assessed outcomes**	**Summary of findings**
Apolinario Vieira et al. (2021) [39]	90 male Wistar Hannover SPF rats 10–12 weeks old	Experimental periodontitis (EP) was induced by placing cotton ligature around the lower 1st molar a. Control b. Ligature c. Ligature + TCZ2 d. Ligature + TCZ4 e. Ligature + TCZ8	Tocilizumab (TCZ)—2, 4, and 8 mg/kg Intraperitoneal injection (daily)	7 and 14 days of TCZ treatment	Micro-CT Histology Gene expression (qPCR)	TCZ reduced alveolar bone loss and attachment loss; diminished inflammatory infiltrate and reduced proinflammatory cytokines
Arabaci et al. (2015) [40]	24 male Wistar rats 12 weeks old (220–250-g body weight)	EP was induced by placing 3-0 silk ligatures around the lower 1st molar for 4 weeks a. Control b. Ligature c. Ligature + MEL	Melatonin (MEL) 10 mg/kg Intraperitoneal injection (daily)	15 days of MEL treatment after ligature removal	Radiographic Histology Biochemical analyses	MEL treatment decreased alveolar bone resorption, MPO and osteoclastic activity
Aral et al. (2015) [41]	72 male Wistar rats (270–330-g body weight)	EP was induced by placing 4-0 cotton ligature around the upper 1st molar for 1 week a. Control b. Periodontitis (P) c. P + SRP d. SRP + vit D_3_ e. SRP + vit K f. SRP + vit D_3_ + vit K	Vitamin K_2_—menatetrenone 30 mg/kg Vitamin D_3_ (2 *μ*g/kg) Oral gavage/daily	10 days of vitamin treatment after ligature removal	Histology Serum levels of ALP and TRAP-5b ELISA (IL-1*β* and IL-10)	Vits D_3_ and K_2_ alone or in combination did not influence the levels of alveolar bone, neither did IL-1*β*, IL-10, ALP, and TRAP-5b levels
Aydinyurt et al. (2021) [42]	30 Wistar albino rats (180–250-g body weight)	EP was induced by placing 4-0 silk ligatures around lower molar for 3 weeks a. SRP b. SRP + iPRF c. i-PRF	Injectable platelet-rich fibrin (i-PRF) Subgingival injection (3 applications)	10 days after ligature removal	Micro-CT Histology IHC	No significant differences were observed among groups regarding bone resorption; inflammation; and TNF-*α*, IL-1*β*, INF-*γ*, and VEGF levels
Cafferata et al. (2019) [43]	C57BL/6 mice 8 weeks old	EP was induced by placing 5-0 silk ligature around the upper 2nd molar a. Control (sham) b. PD group c. PD + 2 *μ*g of IL-35 (ip) d. PD + 1 ng of IL-35 (ig) e. PD + 10 ng of IL-35 (ig) f. PD + 100 ng of IL-35 (ig)	IL-35 daily injections Intragingival (ig) injection of 1, 10, or 100 ng of IL-35 Intraperitoneal injection of 2 *μ*g of IL-35	15 days of IL-35 treatment	Micro-CT Histology ELISA (RANKL and OPG) mRNA expression levels Flow cytometry	IL-35 treatment inhibited alveolar bone loss, decreased osteoclast, and downregulated RANKL expression. IL-35 upregulated Treg-related cytokines and downregulated Th17-related cytokines
Chen et al. (2015) [44]	60 female Sprague–Dawley rats 4 monthsold	Bilateral ovariectomy (OVX) EP was induced by placing 3-0 silk ligature around the upper 1st and 2nd molars for 4 weeks 30 days after OVX a. Control (sham) b. Sham + ligature c. OVX + ligature d. OVX + ligature + Scl − Ab	Sclerostin antibody (Scl-Ab) 25 mg/kg Twice weekly Subcutaneous injection	6 weeks Scl-Ab treatment	Micro-CT ELISA (OCN, TRAP5b and CTx-1) Histology	Treatment of OVX rats with Scl-Ab that underwent ligature-induced PD decreased alveolar bone loss (higher values of mineral apposition rate and mineralizing bone surface). Increased serum OCN and OPG and decreased TRAP and CTx-1 levels
Chen et al. (2017) [45]	50 male Sprague–Dawley rats 6–8 weeks old 180–220-g body weight	Diabetes induced (65 mg/kg streptozotocin (STZ)) EP was induced by placing 4-0 silk ligature around the upper 1st molar 1 day after diabetes confirmation a. Control b. PTH c. Ligature (L) d. L + STZ e. L + STZ + PTH	Parathyroid hormone (PTH) Subcutaneous injection of 75 *μ*g/kg PTH 4 times/week for 4 weeks 1 day after ligature placement	4 weeks of PTH treatment	Micro-CT Histology IHC	Diabetes significantly aggravated alveolar bone destruction induced by ligature placement, and PTH decreased alveolar bone loss and tissue inflammation. PTH increased osteoblastic activity and decreased RANKL/OPG ratio
Cirelli et al. (2009) [46]	45 male Sprague–Dawley rats 8–10 weeks old 200-g body weight	*Pg*-LPS-induced periodontal disease 10 *μ*L of *Pg* W83 LPS (4 palatal gingival sites—a total of 40 *μ*L per animal); injections 3 times/week a. TNFR:Fc b. TNFR : Fc + LPS c. *Pg*-LPS d. Control	TNFR:Fc (100 *μ*L of 1 × 10^11^ DRP) intramuscular administration; 4 weeks before *Pg*-LPS injection	4 and 8 weeks after the first injection	Micro-CT Histology qPCR IHC	TNFR:Fc protected against *Pg*-LPS-mediated alveolar bone loss and reduced the level of proinflammatory cytokines and osteoclast cells in the periodontal tissues
Delima et al. (2002) [47]	9 *Macaca fascicularis* models 3–6 years old	EP was induced by placing silk ligatures inoculated with *P. gingivalis* (strain A7436) around the lower molar a. Control b. Ligature + PBS c. Ligature + sIL − 1R1	Soluble interleukin-1 receptor Type I (sIL-1R1)—6 *μ*g per injection into the gingival tissue around the maxillary molars 3 times weekly	6 weeks	Histology	Inhibition of IL-1 with soluble human IL-1R1 significantly reduced inflammation and bone resorption induced by ligature placement
Gaspersic et al. (2010) [48]	18 female Wistar rats 200–250-g body weight	EP was induced by placing ligature around the upper 2nd molar a. Control b. Ligature 1 week c. Ligature 2 weeks d. Ligature 2 weeks + anti − NGF	Anti-NGF (nerve growth factor) Ab 30 *μ*g Subcutaneous injection	1 and 2 weeks	qPCR IHC X-ray measurement	Systemic anti-NGF treatment reduced IL-1*β* expression and alveolar bone resorption
Goncalves et al. (2014) [49]	Wistar rats 200–250-g body weight	EP was induced by placing 3-0 nylon ligature around the upper 2nd molar a. Control b. Ligature c. Ligature + infliximab	Infliximab (Remicade 100 mg) 5 mg/kg intravenously 1 h before ligature placement	11 days after PD induction	Morphometric analysis Histology MPO Flow cytometry Western blot ELISA (IL-1*β* and TNF-*α*) IHC	Infliximab reduced IL-1*β*, TNF-*α*, and MPO; diminished MMP-1–8, RANK, and RANKL; and attenuated alveolar bone loss
Hu et al. (2017) [50]	C57BL/6 mice 8–10 weeks of age	EP was induced by placing 7-0 silk ligatures around the upper 2nd molar for 14 days	Combination of CD40L (1 *μ*g/mL), IL-21 (1 *μ*g/mL), and anti-Tim1 (5 *μ*g/mL) Interdental papilla injection on Days 3, 6, and 9	2 weeks	qPCR Morphometric analysis TRAP	Combination of IL-21/anti-Tim1/CD40 increased IL-10 gingival mRNA and protein levels and decreased RANKL expression and alveolar bone loss
Jin et al. (2007) [51]	32 male Sprague–Dawley rats 250–300-g body weight	EP was induced by placing 3-0 cotton ligature around the lower 1st molar a. Control b. rhOPG-Fc c. Ligature + rhOPG − Fc d. Ligature + vehicle	Human recombinant OPG fusion protein (rhOPG-Fc) 10 mg/kg subcutaneously Twice a week	3 and 6 weeks	Micro-CT Histology TRAP	OPG-Fc treatment decreased the levels of TRAP-5b, preserved alveolar bone volume, and suppressed osteoclast surface area
Kim et al. (2017) [52]	56 male inbred F344 rats 6 weeks old	Diabetes induced by iv STZ administration EP was induced by placing ligature (dental floss) around the lower 1st molar a. Control b. Periodontitis c. Diabetes + Periodontitis d. Diabetes + periodontitis + IFX	Infliximab (IFX) 5 mg/kg Intraperitoneal; once for the 3-day group (on Day 0) and twice for the 20-day group (Days 7 and 14)	3 days and 20 days after ligature placement	Histology IHC qPCR	IFX treatment demonstrated lower alveolar bone loss, decreased osteoclast formation, and lower RANKL positive osteocytes
Kim et al. (2018) [53]	Male F344 rats	Diabetes induced by iv STZ injection EP was induced by placing ligature (dental floss) around the lower 1st molars a. Control b. Periodontitis (PD) c. PD + PTH d. Diabetes + PD e. Diabetes + PD + PTH	Parathyroid hormone (PTH) administered after ligature placement subcutaneously 3 times per week (40 *μ*g/kg)	30 days	Histology Fluorescence IHC	Rats with diabetes and periodontitis treated with PTH presented with greater osteoid formation, more mineral deposition, lower percentage of sclerostin-positive osteocytes, and diminished alveolar bone loss
Kirkwood et al. (2007) [54]	40 female Sprague–Dawley rats 250-g body weight	EP induced by *Aa* LPS (2 *μ*L of 10 mg/mL solution) injected in the palatal mucosa 3× per week for 8 weeks a. *Aa* LPS b. LPS + SD282 (15 mg/kg) c. LPS + SD‐282 (45 mg/kg) d. Control + vehicle e. Control + SD‐282 (45 mg/kg)	ATP-competitive inhibitor of p38 MAPK SD282 Twice daily by oral gavage	8-week period	Micro-CT Histology IHC TRAP	Both doses of SD282 showed significant protection from LPS-induced bone loss; significantly fewer TRAP-positive osteoclasts; and a significant decrease in IL-6, IL-1*β*, and TNF-*α* expression
Kose et al. (2016) [55]	70 male Sprague–Dawley rats 200–220-g body weight	Diabetes (DM) induced by a single dose of 120 mg/kg alloxan (ip); EP was induced by placing 3-0 silk ligature around the lower 1st molar kept for 4 weeks a. Control b. EP c. DM d. EP + DM e. EP + melatonin f. DM + melatonin g. EP + DM + melatonin	Melatonin (Ketalar, Pfizer) 10 mg/bw Administered by ip injection for 14 days after ligature removal	2 weeks	Biochemical assay MPO activity Histology IHC	Melatonin treatment reduced serum oxidative stress index and alveolar bone loss and decreased MPO activity and osteoclast densities
Kuritani et al. (2018) [56]	C57BL/6 male mice 8 weeks old	LPS (*E. coli* 026:B6) injected into the calvarial bone; EP was induced by placing 5-0 silk ligature around the upper 2nd molar a. LPS (25 mg/kg) b. Anti-RANKL c. Zolendronate d. Control	Anti-RANKL Ab (3 mg/kg) given at 0, 1, and 2 weeks after ligation via ip	2 weeks	Micro-CT TRAP	Anti-RANKL administration inhibited osteoclast formation and bone resorption in calvaria; anti-RANKL also inhibited alveolar bone destruction in the EP mouse model
Lalla et al. (2000) [57]	C57BL/6 male mice 6–7 weeks old	DM induced by 4 ip injections of STZ (55 mg/kg) EP was induced by oral inoculation of *P. gingivalis* (0.2 mL of 1.5 × 10^12^ cells/mL) every other day for a total of 4 days 1 month after DM induction a. Control (no DM) b. DM c. DM + sRAGE	sRAGE at dosage ranging from 3.5 to 100 *μ*g per day. Commencing the day after administration of *P. gingivalis* was completed and continuing for a total of 2 months; daily ip injection	2 months	Morphometric analysis ELISA IHC Immunoblotting MMP protein and activity	sRAGE administration leads to reduced alveolar bone loss in a dose-dependent manner independently of the glycemic level. The levels of MMP-2, MMP-3, and MMP-9; TNF-*α*; and IL-6 were significantly reduced in sRAGE-treated mice
Li et al. (2013) [58]	50 male C57BL/6 WT mice 4 weeks old	DM was induced by ip injection of STZ (40 mg/kg/bw) EP was induced by oral inoculation of *P. gingivalis* (ATCC33277) with 100 *μ*L of 109 CFU of live *Pg* a. Control b. Periodontitis (P) c. DM + P d. P + vit D_3_ e. DM + P + vit D_3_	Vitamin D_3_ hydroxylated to 25-hydroxyvitamin D_3_ (25(OH)D_3_) 5 *μ*g/kg/bw ip injection every other day	8 weeks	ELISA (TNF-*α*) Morphometric analysis Western blot	Administration of 25(OH)D_3_ (ip) reduced fasting glucose and TNF-*α* levels, decreased alveolar bone loss, and attenuated the phosphorylation of Janus family kinase 1 (JAK1)
Li et al. (2019) [59]	30 male C57BL/6 WT mice 6 weeks old 20–228-g body weight	EP was induced by oral inoculation with *P. gingivalis* (ATTC 33277); 3 times at 2-day intervals within 5 days (109 CFU) a. Control b. P c. P + VD_3_	Vitamin D_3_ was ip injected (2.5 *μ*g/kg/bw) every other day starting 5 weeks after oral inoculation, and mice were injected for another 8 weeks	8 weeks	Scanning electron microscopy (SEM) Western blot IHC	Ip injection of vitamin D_3_ for 8 weeks significantly decreased alveolar bone loss. Vit D_3_ decreased NF-*κ*B p65 phosphorylation and NLRP3, caspase 1, IL-1*β*, and IL-6 protein expression
Madeira et al. (2016) [60]	15 C57BL/6 WT mice 8 weeks old	EP was induced by oral inoculation (3 times) with *Aa* (1 × 10^9^ CFU) a. Control b. *Aa* c. *Aa* + DTrp8‐MSH	Melanocortin agonist—DTrp8-gMSH subcutaneously 10 *μ*g/mouse	30 days	Morphometric analysis Histology MPO ELISA Flow cytometry qPCR	Treatment with melanocortin agonist, DTrp8-gMSH, decreased alveolar bone loss; lowered the degree of neutrophil infiltration; and reduced levels of TNF-*α*, IFN-*γ*, and IL-17A
Marques et al. (2009) [61]	76 male Wistar rats 4 weeks old 78 ± 7 g	EP was induced by placing cotton ligature around the lower 1st molar a. Control b. PTH	Human parathyroid hormone (PTH) 40 *μ*g/kg 3×/week subcutaneously 15 days of treatment	15 and 30 days	qPCR Zymography Immunoprecipitation TRAP IHC	PTH treatment decreased MMP-9 activity, decreased osteoclast numbers, and reduced the levels of mRNA for IL-6 and MMP-2
Meng et al. (2014) [62]	31 male C57BL/6 mice 12 weeks old	EP was induced by placing 6-0 silk ligatures presoaked with *P. gingivalis* around the upper 2nd molar a. P b. P + JQ1 c. Control	Bromodomain and extraterminal domain (BET) inhibitor JQ1 (50 mg/kg) Daily ip injection	10 days after ligature placement	Histology qPCR Morphometric analysis	Systemic administration of JQ1 significantly inhibited inflammatory cytokine expression and alveolar bone loss
Nakane et al. (2021) [63]	20 male C57BL/6 mice 8–10 weeks old	EP was induced by placing 6-0 silk ligature around the upper 2nd molar for 5 days a. P b. P + CTLA‐4	Cytotoxic T lymphocyte–associated antigen 4 (CTLA-4) 50 mg/kg ip injection at 1 and 3 days after ligature placement	5 days after ligature placement	Micro-CT Histology TRAP	Systemic administration of CTLA-4 significantly decreased the number of osteoclasts and reduced alveolar bone loss
Napimoga et al. (2018) [64]	18 male mice 20–25-g body weight	EP was induced by oral inoculation with *Aa* (JP2—1 × 10^9^ CFU)—3 inoculations a. Control b. P c. P + TPPU	Soluble epoxide hydrolase (sEH) inhibitor (1-trifluoromethoxyphenyl-3-(1-propionylpiperidin-4-yl) urea (TPPU)) 1 mg/kg/day for 15 days by oral gavage	15 days	Morphometric analysis Western blot PCR array	Systemic treatment with TPPU showed inhibition of alveolar bone resorption, increased expression of sEH, and downregulation of cytokines and molecular markers in the gingival tissue
Pacheco et al. (2021) [65]	15 male BALB/c mice	EP was induced by placing 6-0 silk ligature around the upper 2nd molar a. Ligature b. Ligature + anti − IL − 17 on Day 0 c. Ligature + anti − IL − 17 on Day 2	Anti-IL-17A carried with microparticles (MP) locally delivered into four sites (buccal and palatal gingiva)	8 days after ligature placement	Micro-CT Histology qPCR	Local delivery of anti-IL-17A MP after periodontitis induction inhibited alveolar bone loss and osteoclastic activity and decreased the expression levels of IL-6, an IL-17A target gene
Renn et al. (2018) [66]	56 male Wistar rats 250–300-g body weight	EP was induced by placing 3-0 silk ligature around the upper 2nd molar a. PD + 10 mg melatonin (28 days) b. PD + 50 mg melatonin (28 days) c. PD + 100 mg melatonin (28 days) d. PD + 10 mg melatonin (14 days) e. PD + 50 mg melatonin (14 days) f. PD + 100 mg melatonin (14 days) g. PD h. Control	Melatonin ip injection in the dosage of 10, 50, and 100 mg/kg 14 and 28 days of treatment	4 weeks	Histology ELISA Western blot	Melatonin treatment depressed the TLR4/MyD88-mediated ERK phosphorylation pathway, reduced proinflammatory cytokine levels, decreased the ratio of RANKL/OPG, decreased the extent of bone resorption, and preserved the microstructure and BMD
Repeke et al. (2011) [67]	Male C57BL/6 WT mice 8 weeks old (12 mice/group)	EP was induced by oral inoculation with *Aa* JP2 (1 × 10^9^ CFU) for 3 times a. Control b. PD c. PD + metRANTES 0.05 mg d. PD + metRANTES 0.1 mg e. PD + metRANTES 0.5 mg f. PD + metRANTES 1.5 mg g. PD + metRANTES 5 mg	Specific antagonist of CCR1 and CCR5 receptors—met-RANTES; ip injection of 0.05, 0.1, 0.5, 1.5, and 5 mg/kg on alternative days initiated with PD induction until 30-day postinfection	30 days	Morphometric analysis Flow cytometry ELISA CRP measurement MPO qPCR	At 0.5- up to 5-mg doses, a strong reduction in the alveolar bone loss and inflammatory cell migration were observed. 5-mg dose resulted in the maximum inhibition of inflammatory cell migration. Systemic treatment also downregulated the levels of inflammatory, Th1-type and osteoclastogenic cytokines, and CD3+ and F4/80+ cells
Rogers et al. (2007) [68]	36 female Sprague–Dawley rats	EP was induced by LPS injection of *Aa* (Y4) 2 *μ*L LPS into the palatal gingiva 3 times/week for 8 weeks a. LPS (4 weeks) + SD282 b. LPS (4 weeks) + vehicle c. LPS (8 weeks) d. LPS (4 weeks)	p38 MAPK inhibitor SD282 (45 mg/kg) administered via oral gavage twice daily starting in the fifth week	8 weeks; 4 weeks of treatment	Micro-CT IHC TRAP	Administration of SD282 significantly blocked alveolar bone destruction and significantly reduced IL-1*β*, TNF-*α*, and osteoclast formation
Shi et al. (2020) [69]	C57BL/6 WT mice 6–8 weeks old	EP was induced by oral inoculation with *P. gingivalis* (W83) 1 × 10^10^ CFU in 100 *μ*L performed twice a day for 1 week a. Control b. Periodontitis c. Adoptive transfer	Adoptive transfer of 106 B10 cells by tail vain injection	4 weeks after last oral inoculation	Morphometric analysis TRAP Flow cytometry ELISA qPCR	Transfer of B10 cells alleviated alveolar bone resorption by reducing osteoclastogenesis; increased IL-10, decreased IL-17 and RANKL gene and protein expression; and reduced the proportion of Th17 cells in the gingival tissue
Taut et al. (2013) [70]	Male Sprague–Dawley rats 9–10 weeks old 250–300-g body weight	EP was induced by placing 3-0 silk ligatures around the upper 1st and 2nd molars for 4 weeks and then was removed a. Control + veh (PBS) 2 and 4 weeks b. Control + Scl − Ab 2 and 4 weeks c. EP + veh 3 and 6 weeks d. EP + Scl − Ab 3 and 6 weeks	Sclerostin (Scl-Ab) administered sc at a dosage of 25 mg/kg twice weekly for therapeutic periods of 3 and 6 weeks. Scl-Ab was also locally applied twice weekly for 3 and 6 weeks into the palatal gingiva (5 *μ*L of 35.6 mg/mL solution)	2 weeks 3 weeks 4 weeks 6 weeks	Micro-CT ELISA Histology Fluorescent calcein labeling	Scl-Ab treatment significantly improved maxillary bone healing, as measured by BVF, TMD, and ABL. After 6 weeks of treatment, BVF and TMD values in the Scl-Ab EP group were similar to those in healthy controls. Serum analysis demonstrated higher levels of osteocalcin and PINP
Virto et al. (2018) [71]	42 Wistar rats 8 weeks old 180-g body weight	Obesity induced by HFD. EP was induced by oral inoculation with *P. gingivalis* (W83) and *F. nucleatum* (DMSZ 20482). 1 mL of bacterial suspension (1 × 10^9^ CFU) inoculated for 4 consecutive days during 12 weeks a. HFD + EP b. Normal rats + EP Experimental treatment consisted of SRP + melatonin	Melatonin 25 *μ*g/mL dissolved in drinking water for 4 weeks	3 weeks after treatment initiation	Clinical parameters Luminex Flow cytometry Micro-CT	Melatonin resulted in reduced gingival inflammation and BOP, with reductions in probing depth and enhanced bone repair in the HFD-EP group and significant reduction in proinflammatory cytokines, IL-1*β*, IL-6, MCP-1, and TNF-*α*
Wang et al. (2013) [72]	50 male C57BL/6 WT mice 4 weeks old	Diabetes induced by ip injection of STZ (40 mg/kg) for 5 days. EP was induced by oral inoculation with *P. gingivalis* (ATCC 33277) 109 CFU dispersed in 100 *μ*L, 3 times every other day for 5 days a. Normal control b. EP c. EP + DM c. EP + 25‐OHD_3_ d. DM + EP + 25‐OHD_3_	25-OHD_3_ administered by ip injection at a dose of 5 *μ*g/kg/bw at 2-day interval	8 weeks	ELISA Morphometric analysis IHC Western blot	25-OHD_3_ treatment attenuated DM-EP by reducing serum fasting blood glucose, glycosylated hemoglobin, and TNF-*α* levels, which led to decreased alveolar bone loss. The expressions of Janus family kinase (JAK) 1 and signal transducer and activator of transcription (STAT) 3 as well as their phosphorylation were inhibited
Wu et al. (2022) [73]	DIO (C57BL/6 background)	EP was induced by placing 5-0 silk ligature around the upper 2nd molar for 2 weeks a. EP b. EP + AdipoRon c. EP + AdipoAI	AdipoRon 50 mg/kg/bw AdipoAI 25 mg/kg/bw Oral gavage for 2 weeks concurrently with EP induction	2 weeks	Micro-CT Histology qPCR Western blot Immunofluorescence	AdipoRon and AdipoAI decreased alveolar bone loss and osteoclast number and inhibited the expression of inflammatory markers in the periodontium of DIO animals
Yu et al. (2017) [74]	33 male C57BL/6 WT mice 8–10 weeks old	EP was induced by placing 7-0 silk ligatures around the upper 2nd molar and maintained for 2 weeks a. EP b. EP + CD40L + 1 *μ*M CpG c. EP + CD40L + 10 *μ*M CpG	CD40 ligand (CD40L) and TLR9 agonist cytidine–phosphatase–guanosine oligodeoxynucleotide (CpG) Palatal injections on Days 3, 6, and 9	2 weeks	qPCR Morphometric analysis Histology IHC	CD40L and CpG treatment reduced the IL-10 mRNA expression and the number of IL-10+ CD45+. Alveolar bone loss was decreased, and the gingival expression of IL-1*β*, TNF-*α*, and RANKL was reduced. The number of TRAP-positive cells was also decreased after treatment
Yuan et al. (2011) [75]	34 male C57BL/6 WT mice 12 weeks old	EP was induced by placing 5-0 silk ligature soaked with *P. gingivalis* (WT strain A7436) around the upper 2nd molar a. Vehicle b. EP c. EP + Hu − Fc d. EP + kavain e. EP + OPG − Fc f. EP + RANK − Fc	- Hu-Fc (5 mg/kg, twice/week) - OPG-Fc (5 mg/kg, twice/week) - RANK-Fc (5 mg/kg, twice/week) subcutaneously delivered - Kavain (40 mg/kg, twice/week) intraperitoneal injection Injections were performed at Days 0, 3, and 7	10 days	Histology Cytokine measurement (BioPlex)	OPG-Fc, RANK-Fc and kavain treatment showed significant bone loss reduction. Epithelial downgrowth showed significant reduction in treatment groups with OPG-Fc performing better than kavain or RANK-Fc. Kavain-, OPG-Fc-, and RANK-Fc-treated mice displayed reduced inflammatory cell counts and cytokine expression
Zhang et al. (2014) [76]	Male APN−/− (*n* = 15), DIO (*n* = 10), and WT (*n* = 10) mice	EP was induced by placing 5-0 silk ligature soaked with *P. gingivalis* - APN−/− divided in 3 groups a. EP b. EP + APN c. Control -WT divided in 2 groups a. EP b. Control -DIO divided in 2 groups a. EP b. EP + APN	Adiponectin (APN) - APN administered by systemic infusion (pump delivering 2.5 *μ*g per day) with 1-mg/mL concentration	10 days after EP induction	Morphometric analysis Histology TRAP	Systemic APN infusion reduced alveolar bone loss, osteoclast activity, and infiltration of inflammatory cells in both EP mouse models. Furthermore, adiponectin treatment decreased the levels of proinflammatory cytokines in white adipose tissue of diet-induced obesity mice with EP
Zhang et al. (2020) [77]	36 female Wistar rats 8 weeks old 200–250-g body weight	- EP was induced by placing 3-0 cotton ligature around the upper 2nd molar - Ligatures were removed after 4 weeks a. Control b. EP c. EP + SPL	Superactivated platelet lysate (SPL) - 50 *μ*L of SPL injected locally into the alveolar bone area, below the gingival margin every other day (8 times) for 2 weeks	16 days	Micro-CT Histology qPCR	SPL treatment diminished alveolar bone loss and reduced the gene expression levels of CCL2, CXCL2, IL-6, IL-18, IL-1*α*, IL-1*β*, CXCL10, CXCL16, and CCL5. SPL treatment downregulated NLRP3, AIM2, and CASP1 inflammasome

**(a):** 

**ARRIVE checklist items**	**Study design**	**Sample size**	**Inclusion exclusion criteria**	**Randomization**	**Blinding**	**Outcome measures**	**Statistical methods**
Apolinario Vieira et al. (2021) [39]	1	1	0	1	1	1	1
Arabaci et al. (2015) [40]	1	0	0	1	1	1	1
Aral et al. (2015) [41]	1	0	0	1	1	1	1
Aydinyurt et al. (2021) [42]	1	0	0	1	0	1	1
Cafferata et al. (2019) [43]	1	0	0	1	1	1	1
Chen et al. (2015) [44]	1	0	0	0	0	1	1
Chen et al. (2017) [45]	1	0	0	1	0	1	1
Cirelli et al. (2009) [46]	1	0	0	0	1	1	1
Delima et al. (2002) [47]	1	0	0	0	1	1	1
Gaspersic et al. (2010) [48]	1	0	0	0	1	1	1
Goncalves et al. (2014) [49]	1	0	0	0	0	1	1
Hu et al. (2017) [50]	1	0	0	0	0	1	1
Jin et al. (2007) [51]	1	0	0	0	1	1	1
Kim et al. (2017) [52]	1	1	0	0	1	1	1
Kim et al. (2018) [53]	1	0	0	0	0	1	1
Kirkwood et al. (2007) [54]	1	0	0	0	1	1	1
Kose et al. (2016) [55]	1	0	0	1	0	1	1
Kuritani et al. (2018) [56]	1	0	0	0	0	1	1
Lalla et al. (2000) [57]	1	0	0	0	0	1	1
Li et al. (2013) [58]	1	0	0	1	1	1	1
Li et al. (2019) [59]	1	0	0	1	1	1	1
Madeira et al. (2016) [60]	1	0	0	0	1	1	1
Marques et al. (2009) [61]	1	0	0	0	1	1	1
Meng et al. (2014) [62]	1	0	0	0	0	1	1
Nakane et al. (2021) [63]	1	1	0	1	1	1	1
Napimoga et al. (2018) [64]	1	0	0	0	1	1	1
Pacheco et al. (2021) [65]	1	0	0	0	1	1	1
Renn et al. (2018) [66]	1	0	0	1	0	1	1
Repeke et al. (2011) [67]	1	0	0	0	0	1	1
Rogers et al. (2007) [68]	1	1	0	0	1	1	1
Shi et al. (2020) [69]	1	0	0	0	0	1	1
Taut et al. (2013) [70]	1	0	0	0	0	1	1
Virto et al. (2018) [71]	1	0	0	1	1	1	1
Wang et al. (2013) [72]	1	0	0	1	1	1	1
Wu et al. (2022) [73]	1	0	0	0	0	1	1
Yu et al. (2017) [74]	1	0	0	1	0	1	1
Yuan et al. (2011) [75]	1	0	0	1	1	1	1
Zhang et al. (2014) [76]	1	0	0	1	0	1	1
Zhang et al. (2020) [77]	1	0	0	1	0	1	1

**(b):** 

**ARRIVE checklist items**	**Experimental animals**	**Experimental procedure**	**Results**	**Abstract**	**Background**	**Objectives**	**Ethical statement**
Apolinario Vieira et al. (2021) [39]	1	1	1	1	1	1	1
Arabaci et al. (2015) [40]	1	1	1	1	1	1	1
Aral et al. (2015) [41]	1	1	1	1	1	1	1
Aydinyurt et al. (2021) [42]	1	1	1	1	1	1	1
Cafferata et al. (2019) [43]	1	1	1	1	1	1	1
Chen et al. (2015) [44]	1	1	1	1	1	1	1
Chen et al. (2017) [45]	1	1	1	1	1	1	1
Cirelli et al. (2009) [46]	1	1	1	1	1	1	1
Delima et al. (2002) [47]	1	1	1	1	1	1	1
Gaspersic et al. (2010) [48]	1	1	1	1	1	1	1
Goncalves et al. (2014) [49]	1	1	1	1	1	1	1
Hu et al. (2017) [50]	1	1	1	1	1	1	1
Jin et al. (2007) [51]	1	1	1	1	1	1	1
Kim et al. (2017) [52]	1	1	1	1	1	1	1
Kim et al. (2018) [53]	1	1	1	1	1	1	1
Kirkwood et al. (2007) [54]	1	1	1	1	1	1	1
Kose et al. (2016) [55]	1	1	1	1	1	1	1
Kuritani et al. (2018) [56]	1	1	1	1	1	1	1
Lalla et al. (2000) [57]	1	1	1	1	1	1	1
Li et al. (2013) [58]	1	1	1	1	1	1	1
Li et al. (2019) [59]	1	1	1	1	1	1	1
Madeira et al. (2016) [60]	1	1	1	1	1	1	1
Marques et al. (2009) [61]	1	1	1	1	1	1	1
Meng et al. (2014) [62]	1	1	1	1	1	1	1
Nakane et al. (2021) [63]	1	1	1	1	1	1	1
Napimoga et al. (2018) [64]	1	1	1	1	1	1	1
Pacheco et al. (2021) [65]	1	1	1	1	1	1	1
Renn et al. (2018) [66]	1	1	1	1	1	1	1
Repeke et al. (2011) [67]	1	1	1	1	1	1	1
Rogers et al. (2007) [68]	1	1	1	1	1	1	1
Shi et al. (2020) [69]	1	1	1	1	1	1	1
Taut et al. (2013) [70]	1	1	1	1	1	1	1
Virto et al. (2018) [71]	1	1	1	1	1	1	1
Wang et al. (2013) [72]	1	1	1	1	1	1	1
Wu et al. (2022) [73]	1	1	1	1	1	1	1
Yu et al. (2017) [74]	1	1	1	1	1	1	1
Yuan et al. (2011) [75]	1	1	1	1	1	1	1
Zhang et al. (2014) [76]	1	1	1	1	1	1	1
Zhang et al. (2020) [77]	1	1	1	1	1	1	1

**(c):** 

**ARRIVE checklist items**	**Housing/husbandry**	**Animal care**	**Interpretation scientific implications**	**Generalizability translation**	**Protocol registration**	**Data access**	**Declaration of interest**
Apolinario Vieira et al. (2021) [39]	1	1	1	1	1	0	0
Arabaci et al. (2015) [40]	1	1	1	1	1	0	0
Aral et al. (2015) [41]	1	1	1	1	1	0	0
Aydinyurt et al. (2021) [42]	1	1	1	1	1	0	1
Cafferata et al. (2019) [43]	1	1	1	1	1	0	1
Chen et al. (2015) [44]	1	1	1	1	1	0	1
Chen et al. (2017) [45]	1	1	1	1	1	0	1
Cirelli et al. (2009) [46]	1	1	1	1	1	0	0
Delima et al. (2002) [47]	0	0	1	1	1	0	0
Gaspersic et al. (2010) [48]	0	0	1	1	1	0	0
Goncalves et al. (2014) [49]	1	1	1	1	1	0	0
Hu et al. (2017) [50]	0	0	1	1	1	0	0
Jin et al. (2007) [51]	0	0	1	1	1	0	0
Kim et al. (2017) [52]	1	1	1	1	1	0	0
Kim et al. (2018) [53]	0	0	1	1	1	1	1
Kirkwood et al. (2007) [54]	0	0	1	1	1	0	0
Kose et al. (2016) [55]	1	1	1	1	1	0	0
Kuritani et al. (2018) [56]	0	0	1	1	1	0	0
Lalla et al. (2000) [57]	0	0	1	1	1	0	0
Li et al. (2013) [58]	1	1	1	1	1	0	0
Li et al. (2019) [59]	1	0	1	1	1	0	0
Madeira et al. (2016) [60]	0	0	1	1	1	0	0
Marques et al. (2009) [61]	0	0	1	1	1	0	0
Meng et al. (2014) [62]	0	0	1	1	1	0	0
Nakane et al. (2021) [63]	1	1	1	1	1	1	1
Napimoga et al. (2018) [64]	1	0	1	1	1	0	0
Pacheco et al. (2021) [65]	1	0	1	1	1	0	1
Renn et al. (2018) [66]	0	0	1	1	1	0	0
Repeke et al. (2011) [67]	1	0	1	1	1	0	0
Rogers et al. (2007) [68]	1	0	1	1	1	0	0
Shi et al. (2020) [69]	1	0	1	1	1	0	1
Taut et al. (2013) [70]	1	0	1	1	1	0	0
Virto et al. (2018) [71]	1	1	1	1	1	0	1
Wang et al. (2013) [72]	1	1	1	1	1	0	0
Wu et al. (2022) [73]	1	0	1	1	1	0	1
Yu et al. (2017) [74]	1	0	1	1	1	0	0
Yuan et al. (2011) [75]	1	0	1	1	1	0	0
Zhang et al. (2014) [76]	1	0	1	1	1	0	0
Zhang et al. (2020) [77]	1	0	1	1	1	0	1

## Data Availability

The datasets used or analyzed during the current study are available from the corresponding author upon reasonable request.
